# Generation and Characterization of Human Mesenchymal Stem Cell-Derived Smooth Muscle Cells

**DOI:** 10.3390/ijms221910335

**Published:** 2021-09-25

**Authors:** Srikanth Sivaraman, Jackson Hedrick, Samia Ismail, Chris Slavin, Raj R. Rao

**Affiliations:** Department of Biomedical Engineering, College of Engineering, University of Arkansas, Fayetteville, AR 72701, USA; ss113@uark.edu (S.S.); hedrickjackson19@gmail.com (J.H.); saismail@uark.edu (S.I.); hsslavov@uark.edu (C.S.)

**Keywords:** differentiation, stem cells, smooth muscle cells, vascular disorders

## Abstract

Cardiovascular diseases are the leading cause of death worldwide. A completely autologous treatment can be achieved by using elastogenic mesenchymal stem cell (MSC)-derived smooth muscle cells (SMC) at the affected tissue site of vascular diseases such as abdominal aortic aneurysms (AAA). Thus, our work focused on evaluating the efficacy of (a) the combination of various growth factors, (b) different time periods and (c) different MSC lines to determine the treatment combination that generated SMCs that exhibited the greatest elastogenicity among the tested groups using Western blotting and flow cytometry. Additionally, total RNA sequencing was used to confirm that post-differentiation cells were upregulating SMC-specific gene markers. Results indicated that MSCs cultured for four days in PDGF + TGFβ1 (PT)-infused differentiation medium showed significant increases in SMC markers and decreases in MSC markers compared to MSCs cultured without differentiation factors. RNA Seq analysis confirmed the presence of vascular smooth muscle formation in MSCs differentiated in PT medium over a seven-day period. Overall, our results indicated that origin, growth factor treatment and culture period played a major role in influencing MSC differentiation to SMCs.

## 1. Introduction

As of 2017, vascular diseases contributed to 23.1% of deaths in America, and that percentage is expected to rise due to an increase in the aging population and the number of people diagnosed with comorbid diseases due to cardiovascular ailments [1]. Among vascular diseases, abdominal aortic aneurysms (AAAs) represent dilation and weakening of the abdominal aorta that results from the disruption of the extracellular matrix by chronically overexpressed matrix metalloproteases (MMPs) [2]. In the case of elderly AAA patients, early surgery is ineffective and there are no current active treatments for subcritically sized (<5.5 cm diameter) AAAs [3], leading to 150,000–200,000 deaths per year [4]. Thus far, clinical trials of drug therapies for AAA have all failed [4]. The growth arrest or regression of AAAs is difficult to achieve as auto-regeneration of the elastic matrix is exceedingly limited because of the poor de novo elastic matrix assembly by aneurysmal smooth muscle cells (SMCs) [5], and adult vascular SMCs in general [5].

Previous studies proposed stem cell-based regenerative repair of vascular disorders such as AAAs using MSCs [6]. Results from Hazizume et al. showed that MSCs contributed to the attenuation of AAA in mice through elastin preservation in the aortic wall, associated with inhibited MMP-2 and MMP-9 and inflammatory cytokines, including IL-6, MCP-1, and TNF in vivo [7]. Generation of stem cells with greater elastogenic potential or differentiation towards elastogenic SMC like cells would be ideal to signal aneurysmal SMCs (e.g., via the mediation of secreted growth factors) to coax them toward a more elastogenic, and less activated, phenotype [6]. A translational study by Wang et al. showed that SMCs derived from embryonic (ESC) and induced (IPSC) stem cells could be used to restore urethral sphincter (consisting of primarily SMCs) function [8]. In Rowett nude rats, damaged urethral sphincters were implanted with ESC- and IPSC-derived SMCs. Leak point pressure resembled intact controls at the end of 5 weeks post injection. These results were highly encouraging as the patients’ native bladder smooth muscles had biopsy barriers and were prone to fibrosis. Hence, a novel source of elastogenic SMCs was a welcome finding in repairing the damaged urethral sphincter.

In this context, we looked into previous studies by Swaminathan et al. that demonstrated that rat bone marrow mesenchymal stem cell (BM-MSC)-derived SMC-like cells (BSMC) exhibited significantly higher elastic matrix neoassemblies than aneurysmal rat aortic SMCs (EaRASMCs) and even healthy rat aortic smooth muscle cells (RASMCs) [9]. The study demonstrated that BSMC could stimulate elastic fiber formation and crosslinking and inhibit MMP activity in noncontact co-cultures of the EaRASMCs, which was mediated by their secreted trophic factors [9]. Additionally, studies have shown that tissue-engineered vascular grafts seeded with perinatal stem cell-derived vascular smooth muscle cells aided in arterial replacement, indicating the usefulness of stem cell-derived SMC in correction of congenital heart defects [10]. Hence, we decided to analyze stem cell-derived SMCs as a source of elastic matrix neoassemblies for cardiovascular disorders such as AAAs in this study.

Human MSCs that have capacity for multilineage differentiation [11], are free of the immunogenic and tumorigenic concerns associated with clinical use of pluripotent embryonic SCs (ESCs) and induced pluripotent SCs (iPSCs) [12,13]. Our study focused on differentiating bone marrow-derived stem cells (BMSC) and adipose-derived stem cells (ADSC) into smooth muscle, as BMSC have already been approved by FDA for clinical trials [14] and ADSC has been shown to be safe for use with a serious adverse event rate of only 0.068% [15]. In this study, three different treatments—all-trans retinoic acid (atRA) [16], platelet-derived growth factor-BB (PDGF) [17], and PDGF with transforming growth factor-β_1_ (TGF-β1) [9,18] (previously shown to differentiate BM-MSCs into smooth muscle like cells (SMLC))—were investigated.

Based on earlier findings that stem cell differentiation protocols determined the phenotypes of stem cell-derived SMCs [9], we sought to investigate if differentiation conditions used to alter the phenotype of stem cell-derived SMCs could serve as a metric for selecting cells with high elastogenesis for use in a regenerative therapy for AAAs [19]. While SMCs can exhibit a continuum of phenotypes [20], ranging between the extreme states of (a) synthetic SMCs with high proliferative capacity and (b) fully differentiated contractile SMCs, which primarily enable tissue contraction, these states are not mutually exclusive. Our study focused on identifying the best protocol to differentiate stem cells into more contractile SMCs.

Finally, we had to consider that SC differentiation capacity can be influenced by donor diversity. Previous studies have shown that cell yield and proliferation have been influenced by external factors like age, gender, or status of health in other cell types [21,22,23]. Studies have also shown the effects of aging on stem cell function [24]. Recently, it was demonstrated that a decline in the size of the progenitor pool hampered the ability of stem cells to repair aged muscle [24].

Apart from studying the effects of age, differentiation factors and time periods, we compared the RNA composition in cells cultured in differentiation medium (DM) with cells grown in cell culture medium (CCM). In particular, we studied the upregulation of long-coding RNA (lnCRNA), micro RNA (miRNAs) and circular RNA (circ RNA) related to smooth muscle in the various cell lines subjected to differentiation treatments. 

Thus, the overall objective of our study was to identify (a) the quickest and most efficient method to differentiate stem cells into smooth muscle cells, and (b) the effects of differentiation factors and origin of stem cells in the differentiation process. We also looked at changes in RNA composition during differentiation of stem cells into smooth muscle, specifically the upregulation of specific SMC gene markers. This research thus became the first study to compare various differentiation factors and mesenchymal stem cell lines to determine the most relevant stem cell line, differentiation factor, and time period combination for obtaining SMCs.

## 2. Results

### 2.1. MSCs Cultured in PDGF and TGF β1 Supplemented Differentiation Medium Indicates SMC Formation 

**Figure 1:**
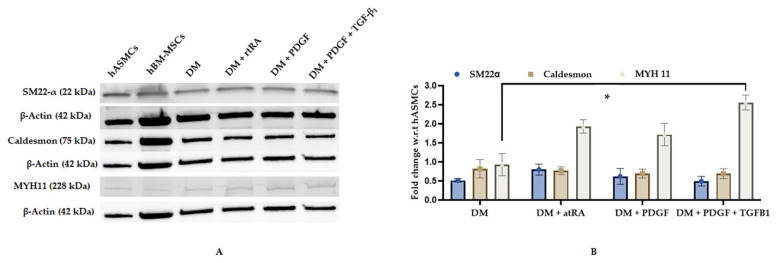
Western Blot Analysis for various SMC markers of M3 cell linesubjected to different differentiation medium (DM) treatments. hBM-MSCs were plated onto fibronectin-coated T175 flasks and treated for four days with DM, DM with all-trans retinoic acid (DM + atRA), DM with platelet-derived growth factor-BB (DM + PDGF), and DM with PDGF and transforming growth factor-β1 (DM + PDGF + TGFB1), respectively. Western blot analysis was performed for smooth muscle cell markers (**A**) SM22-α, caldesmon, and smooth muscle myosin heavy chain-11 (MYH11). Fold change was determined with respect to DM treatment (**B**). The treatment (DM + PDGF + TGFB1) showed a statistically significant increase in MYH11 (B) expression (*-*p*-value < 0.05).

**Figure 2:**
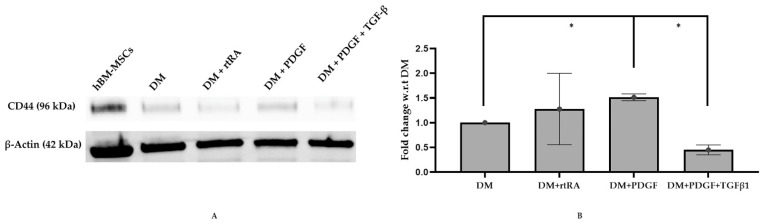
Western blot analysis of MSC marker of M3 cell line subjected to different DM treatments. Treated hBM-MSCs were plated onto fibronectin-coated T175 flasks and treated for 4 days with DM, DM + atRA, DM + PDGF, and DM + PDGF + TGF-β1. Western blot analysis was performed for stem cell marker CD44 (**A**) and normalized to β-actin. Fold change was determined with respect to DM treatment (**B**). The treatment supplemented with PDGF and TGF-β1 (DM + PDGF + TGF-β1) showed a statistically significant decrease in CD44 expression when compared to DM (*-*p*-value < 0.05) (**B**). The treatment supplemented with PDGF (DM + PDGF) showed a significant increase in CD44 expression when compared to DM (*-*p*-value < 0.05) (**B**).

M3 (bone marrow mesenchymal stem cells derived from a 21-year-old male Asian) cell lines, after four days in cell culture medium (CCM), exhibited early and mid-stage SMC markers (SM22α and caldesmon) while differentiation medium (DM), supplemented with PDGF and TGF-β1, resulted in a statistically significant increase in MYH11 (late stage smooth muscle marker) (Figure 1) and a significant decrease in CD44 (stem cell marker) (Figure 2). M3 cell line, cultured in DM supplemented with trans RA, exhibited the highest expression of MYH II and low expression of SM22 α and caldesmon (Figure 1). However, it did not express a statistically significant decrease in CD44 expression (Figure 2). Similarly, M3 cell line, cultured in DM supplemented with PDGF, exhibited the highest expression of MYH II and low expression of SM22 α and caldesmon (Figure 1), but recorded an increase in CD44 expression (Figure 2). Hence, we probed our samples for MYH11 in our flow cytometry analyses, as it conclusively established contractile smooth muscle cell formation.

### 2.2. Treatment of MSCs with DM Containing PT Resulted in Minimal Increase in MYH II Expression

Similar to our Western blotting results, the flow cytometry results indicated increased MYH II expression in PT treated cultures compared to tRA and PDGF treated cultures. The cell lines cultured for 14 days in DM medium (tRA/PDGF/PT) did not survive at the end of 14 days. The FlowJo data analysis indicated that in the M1 cell line, either the four-day or seven-day differentiation with growth factors (PDGF + TGFβ1) could show higher MYH11 expression than M1 cultured in ATCC CCM or DM with other growth factors (trans RA, PDGF) (Table 1).

**Figure 3:**
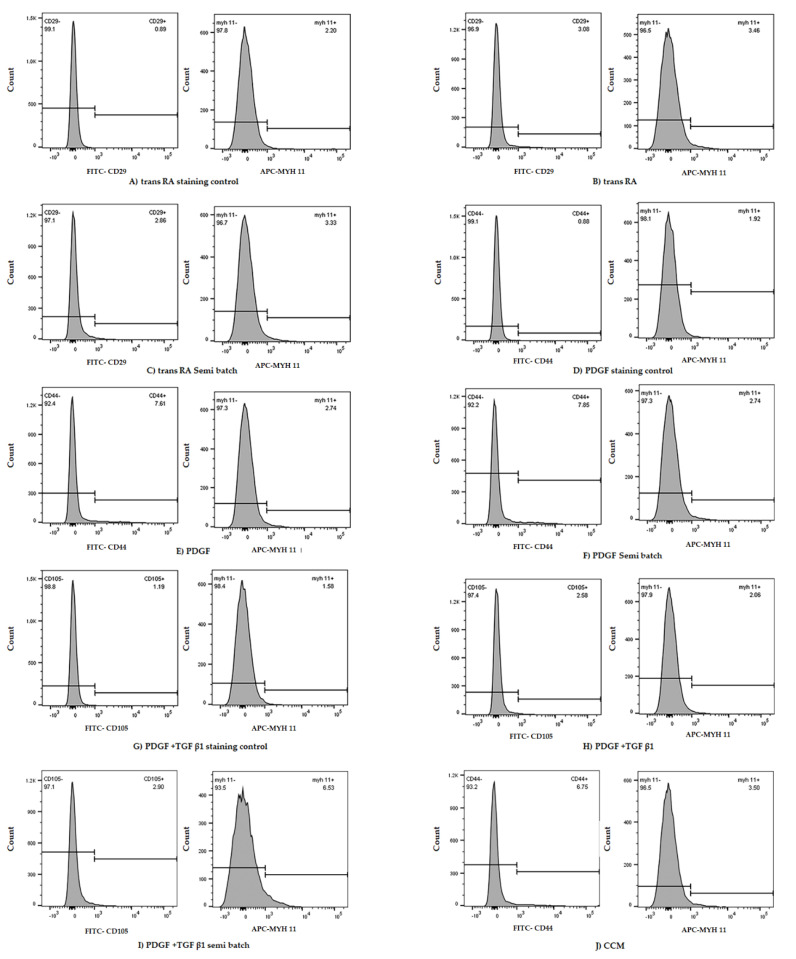
Representative flow cytometry data from M1 cell line subjected to 7 days in different DM treatments (**A**–**J**). Staining control samples were subjected to only secondary antibodies (**A**,**D**,**G**). Each non-control tube was stained with two primary antibodies which denoted SMC (MYH II) and MSC (CD29, CD44, CD105) markers, respectively (**B**,**C**,**E**,**F**,**H**,**I**). The cell line cultured in cell culture medium (CCM) is considered an experimental control. In this trial, the samples subjected to PDGF and TGF β1 (semi-batch) (**I**) elicited the highest MYH11 expression.

After 7 days of differentiation in PDGF + TGFβ1 treatment, the MYH11 expression of the M1 cell line was double the expression of M1 cultured in ATCC CCM (Figure 3). Similarly, in the M2 and M3 cell line, there was a trend toward increases in MYH11 expression after four days in PDGF + TGFβ1 (PT) semi-batch treatment and PDGF + TGFβ1 (PT) treatment, respectively, compared to M2 and M3 in the CCM and other growth factor (trans RA, PDGF) conditions (Table 1).

**Figure 4:**
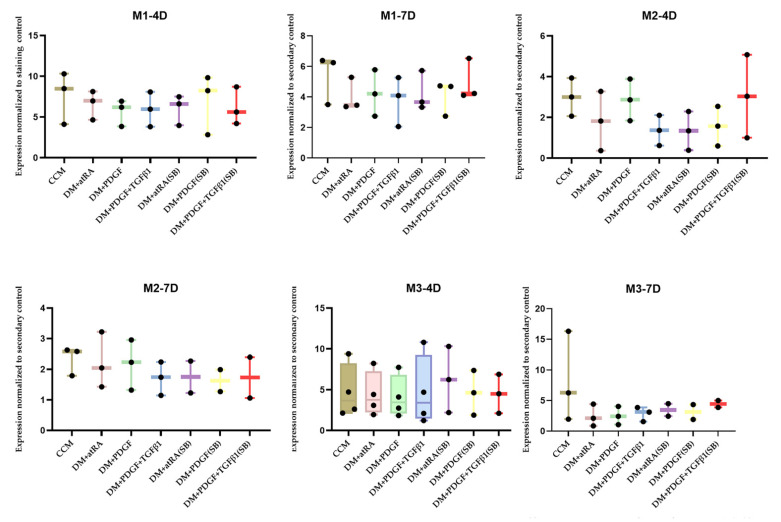
Comprehensive flow cytometry data from human bone marrow (hBM)-MSC cell lines subjected to different DM treatments and time points (n = 3 per cell-line/growth-factor treatment/timepoint) and analysis of MYH II expression. The treatments without primary antibody addition are considered secondary controls. The differences between the treatments were not statistically significant. Each data point was obtained based on the measurement of 20,000 events in FACS CANTO II.

**Figure 5:**
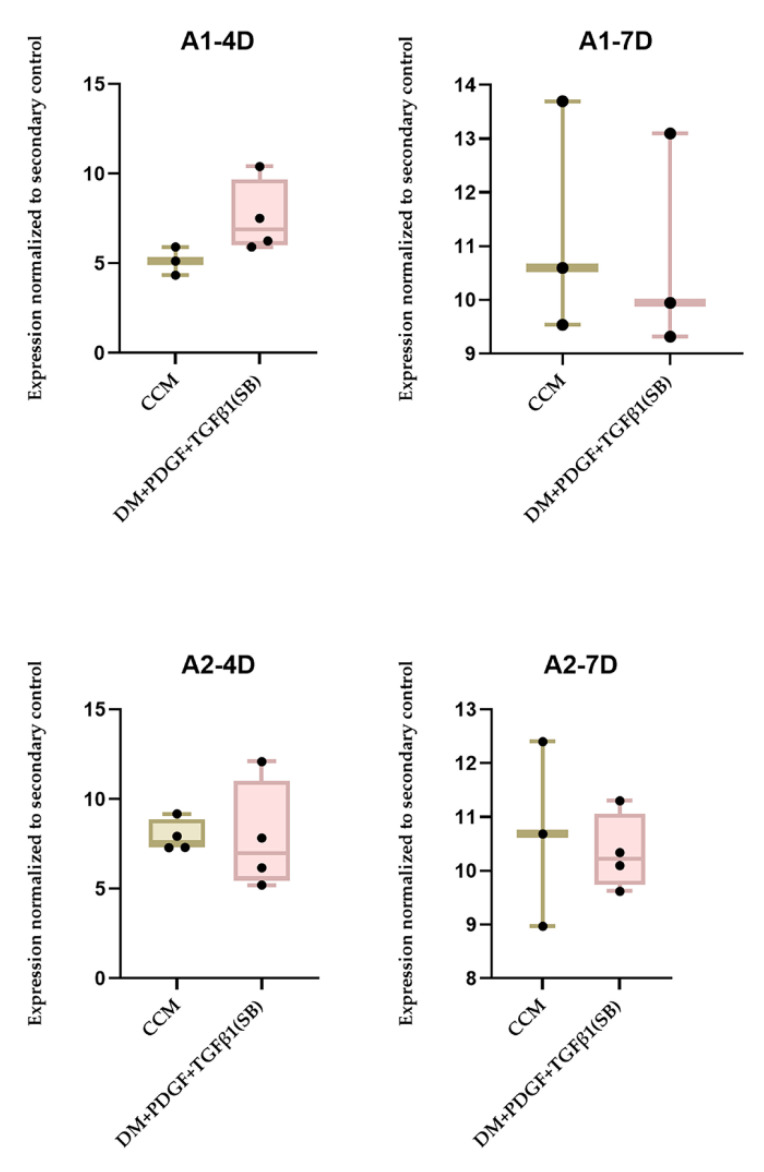
Comprehensive flow cytometry data from human adipose (AD)-MSC cell lines (A1,A2) subjected to PT supplemented DM semi-batch culture at four-(A1–4D,A2–4D) and seven-(A1–7D,A2–7D)day time points (n = 3 per cell-line/treatment/timepoint) and analysis of MYH II expression compared to cell lines cultured in CCM (control). The treatments without primary antibody addition are considered secondary controls. The differences between the treatments were not statistically significant. Each data point was obtained based on the measurement of 20,000 events in FACS CANTO II.

However, the difference in MYH11 expression was not significant in any of these cases (Figure 4). The results from the FlowJo data analysis for ADSC differentiation to SMC (4, 7 days) indicated that, although the PT semi-batch treatment could elicit relatively higher SMC expression than ADSC grown in CCM (Table 2), the differences were not significant (Figure 5).

### 2.3. Origin of Stem Cells and PT Treatment Both Play a Role in MSC Differentiation to SMC

The RNA Seq analysis in PT-induced ADSC indicated that (a) TAGLN was upregulated in A1PT (adipose stem cells derived from a 24-year-old female caucasian) compared to A1CC and (b) ACTA-2 was upregulated in A2PT (adipose stem cells derived from a 29-year-old female native American) compared to A2CC (Table 3). The microRNA analysis indicated that (a) miRNA 145-5p was upregulated in A2PT, compared to A2CC and (b) miRNA 21-5p was upregulated in A1PT, compared to A1CC (Table 3).

Circ RNA data from Novogene indicated the upregulation of more vascular smooth muscle-specific circ RNA in A2PT, such as CALD 1 and PP1R12C, compared to cells cultured in A1PT (Table 4)

**Figure 6:**
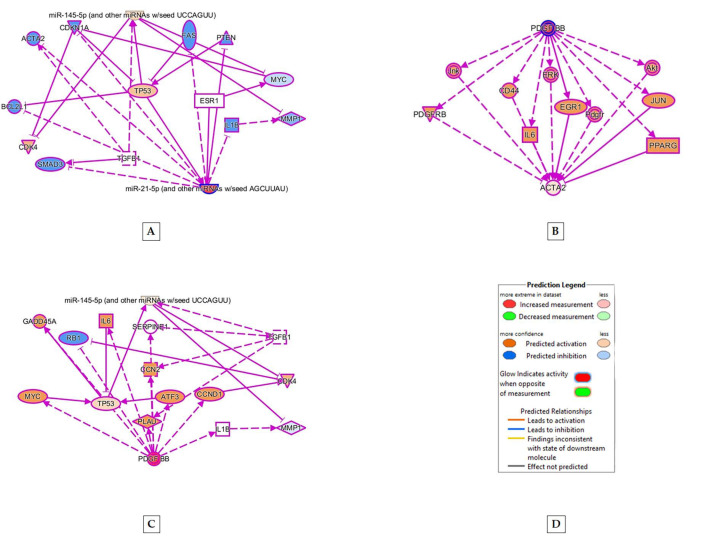
Molecular upregulation and pathway analysis between (**A**) miRNA 21-5p and miRNA 145-5p, (**B**) PDGF and miRNA 145-5p, and (**C**) PDGF-BB and ACTA2 using ingenuity pathway analysis (IPA). (**D**) represents the color code denoting up/downregulation of molecules in the pathways (**A**–**C**). Pathway (**A**) shows that miRNA 21-5p upregulates miRNA 145-5p. The analysis of (**B**) indicates that PDGF and TGFβ1 independently upregulates miRNA 145-5p. The pathway (**C**) indicates that PDGF-BB upregulates ACTA2.

IPA analysis between various smooth muscle-specific RNA indicated that pathways between mRNA such as ACTA2 (expressed in smooth muscle), miRNA 21-5p (indicates stem cell differentiation) and 145-5p (expressed in vascular smooth muscle cells) indicated involvement of TGFβ1 in upregulating smooth muscle-related MRNA and miRNA (Figure 6). In addition, PDGF independently upregulated ACTA 2 and miRNA-145-5p (Figure 6).

## 3. Discussion

Through the use of Western blot analysis, we examined the SMC differentiation after four days of growth factor treatment of hBM-MSCs. hBM-MSC basal expression of SMC markers such as SM22-α and caldesmon were present at levels similar to that found in human aortic SMCs (hASMCs) (Figure 1). However, hBM-MSC basal expression of the late-stage marker, MYH11, was significantly less than in hASMCs (Figure 1). This evaluation was congruent with that shown by Tamama et al. in which untreated hBM-MSCs cultured in CCM expressed early- and mid-stage SMC markers, α-smooth muscle actin and caldesmon, with minimal expression of smooth muscle myosin heavy chain [25]. Moreover, the ATCC cell culture medium with growth kit contained growth factors, such as rhFGF basic and rhIGF-1, which can induce early-stage smooth muscle markers such as SM22-α. Therefore, it was critical for further experiments to only include late-stage SMC markers such as MYH11 in the evaluation of hBM-MSC differentiation into SMCs, in order to accurately detect SMC formation.

The Western blot analysis also showed that (Figure 2), treatment with differentiation medium (DM) supplemented with PDGF and TGF-β_1_ (PT) for four days showed a statistically significant increase in MYH11 expression and a significant decrease in CD44 expression over other treatments. Treatment with DM, supplemented with PDGF, revealed an increase in CD44 expression. Based on these results, treatment of hBM-MSCs with DM containing PT treatment was the most relevant method to obtain vascular smooth muscle. Our pathway analysis indicated that PDGF upregulated ACTA2 (expressed in smooth muscle), and PDGF and TGFβ1 independently upregulated miR-145, expressed in vascular smooth muscle (Figure 6).

Similarly, flow cytometry analysis indicated that, in two of the three bone marrow mesenchymal stem cell lines (M1 and M2), the semi-batch PT treatment for four days seemed to elicit the highest expression of MYH11 among all treatments (Table 1). M3 showed the highest MYH11 expression in the non-semi-batch PT treatment (Table 1) among all treatments. However, the MYH11 expression differences were not statistically significant between treatments (Figure 4). These results were similar to results shown in studies by Dahal et al. where rat BM-SMCs generated in the presence of TGF-β1 with PDGF-BB (21 day culture) exhibited lower expression of MYH 11 expression relative to RASMCs and expression levels of key SMC marker proteins (caldesmon, a-SMA, smoothelin, and MHC) were not significantly different between the cell groups [26]. However, based on the Western blotting and hBM-MSC flow cytometry analysis, only PT treatments could elicit smooth muscle like cells and hence, we only looked at PT semi-batch treatment in subsequent experiments.

Both A1 and A2 cell lines showed higher MYH 11 expression in PT-treated medium than CCM-treated cell lines (Table 3), but the differences were not statistically different (Figure 5). The IPA analysis of RNA Seq data indicated that PT treatment caused human adipose stem cell differentiation into smooth muscle-like cells (Table 3 and Table 4). There was more propensity for stem cell differentiation into smooth muscle in A2PT compared to A1PT (Table 3 and Table 4), indicating the importance of cell source in differentiation.

Our results preliminarily denoted that AD stem cells from older sources (A2) showed more vascular smooth muscle-specific LnC RNA, miRNA and circ RNA than AD-MSC from relatively younger patients (A1). This was similar to findings from Krawiec et al. who showed that age was critically important to both the abilities of AD-MSCs to produce secreted factors and differentiate into SMCs [27] and gender. BMI did not seem to play a role in either stem cell function.

RNA Seq data analysis also showed that miRNA 17-5p was expressed in A2 cell lines cultured in PT infused differentiation medium, compared to A2 cell lines cultured in cell culture medium. MiRNA17-5p expression is associated with adipose stem cell differentiation [28] and hence, indicates the successful differentiation of the A2 cell line in seven days of PT treatment. Several microRNAs were found to be related to SMC differentiation during the development and the maturation of vascular SMC phenotypes, such as miR-143/145 [29,30]. Additionally, miR-143/145 were upregulated upon TGFβ1 treatment, which suggested the interaction of miRNAs and conventional signaling pathways in SMC phenotype switching [31]. In vitro, these miRs augmented SMC differentiation and maturation under certain conditions [29]. Our RNA-Seq data analysis showed that miR-145-5p was upregulated when A2PT was compared with A1CC (Table 3), indicating vascular SMC differentiation. Furthermore, miRNA 145 was not upregulated in A1PT, showing the relevance of stem cell source in quicker vascular smooth muscle formation. The IPA analysis of RNA-Seq data showed that miR-21 was upregulated when A1PT was compared with A1CC, and also when A2PT was compared with A1PT, indicating that AD-MSC had differentiated in both cases (Table 3). In addition to promoting contractile gene expression in response to TGF-β, miR-21 was found to promote vSMC proliferation and reduce apoptosis [32]. Based on our pathway analysis, miR-21 mildly activated miR-145 and thus, promoted stem cell progression towards differentiation to vascular smooth muscle (Figure 6A).

Our circ RNA analysis from RNA-Seq data indicated that ROCK2 genes were upregulated in A2PT compared to A2CC (Table 4). Previous studies have shown that the ROCK pathway could be activated upon TGFβ1 stimulation and has been known to participate in SMC differentiation [33]. There are two isoforms of ROCK, including ROCK2, that are mainly expressed in SMC [34]. When TGFβ1 was used to stimulate MSC, the level of miR222-5p was downregulated in the differentiation process, resulting in derepression of its direct targets (ROCK2) and subsequent promotion of SMC differentiation [34]. Similarly, studies by Alimperti et al. implicated adherens junctions, specifically cadherin-11, in the development of the contractile phenotype of SMCs through ROCK isoforms such as ROCK1 and ROCK2 [35]. However, ROCK genes were not upregulated when A1PT was compared to A1CC. Thus, the data indicated that the origin (Native American) of stem cells and the application of PT treatment played a role in MSC differentiation to SMC.

**Figure 7:**
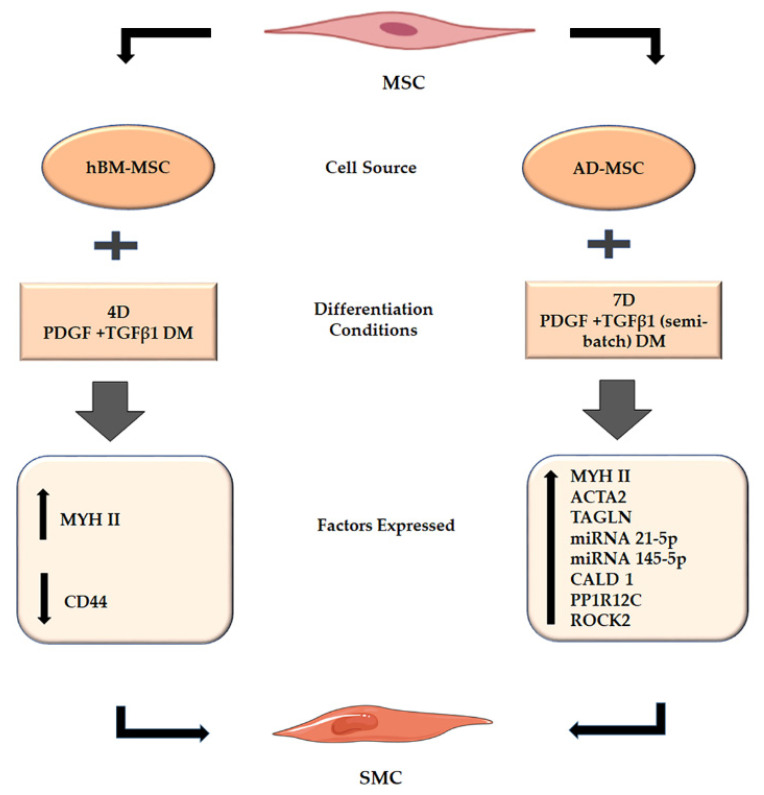
Summary figure describing the MSC differentiation into SMC in our study. PT treatment (batch and semi-batch) on both time points (4Days, 7Days) could elicit various cell markers (hBM-MSC) and RNA sequences (AD-MSC) representative of SMC formation. The cell marker analysis indicated the upregulation in smooth muscle (MYH II) markers and downregulation of stem cell (CD44) markers after PT treatment. The RNA Seq analysis indicated that PT treatment caused upregulation of RNA associated with vascular smooth muscle cells.

Thus, the results from our research (Figure 7) indicated that, among the various treatments, the PT treatment was most effective in differentiating mesenchymal stem cells derived from human bone marrow and adipose tissue into smooth muscle cells. Both PDGF and TGFβ1 independently upregulated RNA sequences associated with vascular smooth muscle cells.(Figure 6). Our study also indicated the importance of origin or cell source in differentiation, as certain cell lines (A2) could express more vascular smooth muscle-related RNA (Table 3) than others (A1).

This study was aimed at evaluating the efficacy of (a) the combination of various growth factors, (b) different time periods and (c) different MSC lines to determine the treatment combination which generated SMCs that exhibited the greatest elastogenicity among the tested groups. However, our study was limited to analysis using bone marrow- and adipose-derived stem cells. Future studies could also evaluate the potential of human embryonic and induced pluripotent stem cells to gain a more comprehensive understanding of the potential to derive elastogenic SMCs from different stem cell sources.

## 4. Materials and Methods

### 4.1. Procurement of Mesenchymal Stem Cells

The adipose-derived mesenchymal stem cell lines—(A1: 45-year-old female African American), (A2: 24-year-old female Caucasian), and (A3: 29-year-old female Native American)—were obtained from the University of Missouri. Two bone marrow-derived mesenchymal stem cell lines—(M1: 30-year-old female Hispanic), (M2: 21-year-old male Asian)—were purchased from ATCC (Manassas, VA, USA), and one bone marrow-derived mesenchymal stem cell line (M3: 18-year-old black female) was purchased from Lonza (Walkersville, MD, USA).

### 4.2. Directed Differentiation of MSCs into SMCs

The directed differentiation of MSC to SMCs was done based on previous methods [9]. Briefly, human bone marrow mesenchymal stem cells (hBM-MSCs; Lonza, MD, USA) were seeded on tissue culture polystyrene flasks (USA Scientific, Ocala, FL, USA) at a density of 2 × 10^3^ cells/cm^2^ in low-glucose DMEM medium (Gibco, Waltham, MA, USA), supplemented with 10% *v*/*v* MSC qualified FBS (Gibco, Waltham, MA, USA) and 1% *v*/*v* antibiotic/antimicotic (AB/AM) (Gibco, Waltham, MA, USA). Human bone marrow mesenchymal stem cells (hBM-MSCs; Manassas, ATCC VA, USA) were seeded on tissue culture polystyrene flasks (USA Scientific, Ocala, FL, USA) at a density of 2 × 10^3^ cells/cm^2^ in mesenchymal basal medium (ATCC^®^ PCS-500-030™) supplemented with mesenchymal stem cell growth kit (ATCC^®^ PCS-500-041™). All the cell lines were seeded in human fibronectin (hFN, 100 ng/mL Gibco, Waltham, MA, USA)-coated T25 tissue-culture flasks (Cell star, Greiner Bio-One, Monroe, NC, USA) at a density of 2 × 10^3^ cells/cm^2^ in differentiation medium (DM) containing 60% *v*/*v* low-glucose DMEM (Gibco, Waltham, MA, USA), 40% *v*/*v* MCDB-201 (Sigma-Aldrich, St. Louis, MO, USA), 1% *v*/*v* AB/AM (Gibco, Waltham, MA, USA), 1× insulin–transferrin–selenium (ITS; Gibco, Waltham, MA, USA), 1× linoleic acid bovine serum albumin (LA-BSA; Sigma-Aldrich, MO, USA), 10^−4^ M ascorbic acid (Sigma-Aldrich, St. Louis, MO, USA), 10^−9^ M dexamethasone (Sigma-Aldrich, MO, USA), 10 ng/mL mouse epidermal growth factor (EGF; Sigma-Aldrich, St. Louis, MO, USA), 103 U/mL mouse leukemia inhibitory factor (LIF; Sigma-Aldrich, MO, USA) and supplemented with 15µg/mL trans RA (Abcam, Cambridge, UK) (DM1), or 10 ng/mL platelet-derived growth factor (PDGF; Sigma-Aldrich, St. Louis, MO, USA) (DM2) or a combination of 10 ng/mL platelet-derived growth factor (PDGF; Sigma-Aldrich, St. Louis, MO, USA) and 2.5 ng/mL transforming growth factor β1 (TGFβ1; EMD Millicorp, Darmstadt, Germany) (DM3) in different flasks for 4, 7 or 14 days of differentiation. Cell lines grown in cell culture medium (CCM) were used as control. The differentiation medium was changed every alternate day in a batch as well as semi-batch manner, and the cells were maintained in 5% CO_2_ at 37 °C.

### 4.3. Western Blot Assessment of Cell Differentiation

M3 cell line was subjected to four days of differentiation in five kinds of medium formulations and the cells at the end of the experiment were subjected to BCA protein assay (Thermo Scientific, Waltham, MA, USA).

### 4.4. BCA Protein Assay

A bicinchoninic acid (BCA) protein assay (Thermo Scientific, Waltham, MA, USA) was used to quantify the protein concentrations from each sample. Samples were diluted ten-fold with Pierce RIPA lysis buffer (Thermo Scientific, Waltham, MA, USA). Standards were created for the following concentrations using bovine serum albumin (Thermo Scientific, Waltham, MA, USA): 2000, 1500, 1000, 750, 500, 250, 125, 25, and 0 μg/mL. RIPA lysis buffer (Thermo Scientific, Waltham, MA, USA) was used as the diluent for the protein standards. A 25 μL aliquot of each standard and diluted sample was added in duplicate to a 96-well microplate, and 200 μL of Pierce BCA protein assay reagent mix (Thermo Scientific, Waltham, MA, USA) was added to each standard and sample. The microplate was covered and incubated for 30 min at 37 °C. The absorbance at 562 nm was measured for both the standards and the samples using a BioTek Synergy 2 plate spectrophotometer and BioTek Gen5 software. The absorbance values and the known concentration of the standards were used in Microsoft Excel to generate a quadratic regression. This standard curve was then used to evaluate the unknown protein concentration of the samples.

### 4.5. Western Blotting

#### 4.5.1. Sample Preparation

Samples were mixed with 2× Laemmli sample buffer (Bio-Rad, Hercules, CA, USA) containing 2-mercaptoethanol (Aldrich Chemistry St. Louis, MO, USA) at a 1:1 ratio and vortexed. The samples were then boiled at 95 °C for 5 min using a water bath and vortexed. The samples were centrifuged at 16,000× *g* for 1 min.

#### 4.5.2. Gel Electrophoresis

A 4–20% Mini-PROTEAN TGX Stain-Free protein gel (Bio Rad, Hercules, CA, USA) was prepared with Tris/glycine/sodium dodecyl sulfate (SDS) buffer (Bio-Rad, 25 mM Tris, 192 mM glycine, 0.1% SDS) for gel electrophoresis. The gel was loaded with Precision Plus Protein Western C standard (Bio-Rad, Hercules, CA, USA) and the samples were equilibrated for total protein content. The gel was then electrophoresed with a Mini-PROTEAN Tetra System (Bio-Rad) and PowerPac Basic (Bio-Rad, Hercules, CA, USA).

#### 4.5.3. Transfer

Following electrophoresis, proteins were transferred onto polyvinylidene difluoride (PVDF) membrane (Bio-Rad, Trans-Blot Turbo Transfer Pack, Hercules, CA, USA) using a Trans-Blot Turbo Transfer System (Bio-Rad, Hercules, CA, USA). After transfer, the membrane was submerged in superblock blocking buffer (Thermo Scientific, Waltham, MA, USA) for one hour at room temperature.

#### 4.5.4. Blotting

Primary antibodies such as stem cell marker CD44 (R&D Systems), early-stage SMC marker SM22-α (Abcam, Cambridge, UK), mid-stage SMC marker caldesmon (Sigma-Aldrich), late-stage SMC marker smooth muscle myosin heavy chain-11 (MYH11, Abcam, Cambridge, UK), and loading control β-actin (Sigma-Aldrich, St. Louis, MO, USA) were incubated at room temperature for 1 h or overnight at 4 °C. After primary antibody incubation, the membrane was washed 3× for 5 min in Tris-buffered saline (Bio-Rad, Hercules, CA, USA) with 0.1% Tween-20 (Fisher Scientific, Hampton, NH, USA) (TBST). Following the washes, respective horse-radish-peroxidase-conjugated secondary antibody was applied to the membrane. Secondary antibodies included: goat anti-rabbit IgG (Abcam, Cambridge, UK), rabbit anti-goat IgG (Abcam, Cambridge, UK), and goat anti-mouse IgG (Abcam, Cambridge, UK).

#### 4.5.5. Imaging

After three more washes with TBST, a stain-free blot image was acquired using a ChemiDoc Imaging System (Bio-Rad, Hercules, CA, USA) and used for total protein normalization. The membrane was then incubated for five minutes in chemiluminescent substrate (Clarity or Clarity Max Western ECL Blotting Substrate, Bio-Rad, Hercules, CA, USA). Chemiluminescent blot images were captured using a ChemiDoc Imaging System (Bio-Rad, Hercules, CA, USA).

#### 4.5.6. Blot Stripping

Blot antibodies were removed by applying a low pH stripping buffer (0.20 M glycine, 3.47 mM SDS, 1% Tween-20, pH 2.2) twice for 10 min each. The membrane was then washed 2× with PBS (Hyclone, UT, USA) for 10 min followed by two TBST washes for 5 min. The membrane was then blocked and reprobed with antibody when necessary.

#### 4.5.7. Protein Normalization

Utilizing Image lab software (Bio-Rad, Hercules, CA, USA), blot pixel intensity for each protein band of interest was quantified and normalized to the stain-free blot image. For the instances in which a stain-free blot image was incompetent, blots were stripped and reprobed for the housekeeping protein, β-actin, and subsequently normalized to β-actin.

### 4.6. Intracellular and Extracellular Marker Staining of Cells for Flow Cytometry

The cells, after being subjected to differentiation, were stained with intracellular and extracellular markers based on previous methods [36]. Briefly, after the differentiated cells were harvested using TrypLE express enzyme (Gibco, Waltham, MA, USA), the cells were added to 15 mL centrifuge tubes. The cells were then washed twice with 3 mL of PBS by centrifuging at 200× *g* for four minutes. Next, the cells were then fixed with 4% PFA for 10 min and washed subsequently with 3 mL of PBS twice by centrifuging at 400× *g* for four minutes. The cells were blocked with blocking solution (6% goat serum/94% PBS) for 45 min. The tubes were covered in foil followed by the addition of primary antibody CD29+/44+/105+ made in blocking solution and incubation for 1 h at room temperature. The cells were subsequently washed in 3 mL of blocking solution twice, followed by centrifugation at 400× *g* for four minutes. The cells were incubated in secondary antibody (Alexa fluor 488, Abcam, Cambridge, UK) for 1 h at room temperature (or overnight at 4 °C). This was followed by washing of the cells in 3 mL of blocking solution twice by centrifuging at 400× *g* for four minutes. The cells were permeabilized by adding 3 mL of permeabilization solution (50 mL of high salt buffer with 25 µL of Tween 20) thrice and centrifuged at 400× *g* for four minutes followed by addition of second primary antibody (Myosin heavy chain 11(Myh 11), Abcam, Cambridge, UK) made in 6% goat serum and incubation for 1 h at room temperature. The cells were washed in 3 mL of blocking solution twice, followed by centrifuging at 400× *g* for four minutes. Subsequently, the secondary antibody (CF 647, Sigma-Aldrich, MO, USA) was added and the cells were incubated for 1 h at room temperature (or overnight at 4 °C). Finally, the cells were washed in 3 mL of blocking solution twice and centrifuged at 400× *g* for four minutes. Samples with no primary antibody addition were used as staining (secondary) controls. Lastly, the samples were analyzed by flow cytometry or were alternatively stored at 4 °C for future testing.

### 4.7. Flow-Cytometry Assessment of Cell Differentiation

Expression levels of stem cell and SMC-specific markers on BM/AD-SMCs were analyzed and compared to control cell types (BM/AD-MSCs in CCM) using flow cytometry [36,37,38]. Briefly, the marker treated cells in 1 mL of blocking solution were added to a glass vial. The cells were then analyzed using a BD-FACS CANTO II flow cytometer (BD); 20,000 events were collected from each experimental group and the data were analyzed using FlowJo^®^ software. Each cell line was differentiated in different growth factor conditions in triplicate.

### 4.8. RNA Seq Analysis of Cell Differentiation

#### 4.8.1. Stem Cell Differentiation and Pellet formation

Two adipose-derived stem cell lines (24-year-old female Caucasian, 29-year-old female Native American) were differentiated in PDGF + TGFβ1 (semi-batch)-induced differentiation medium for seven days. The two cell lines, cultured in their respective cell culture medium, were treated as controls. Each flask was seeded with 50,000 stem cells during the experiments. After the 7-day differentiation time point, the cells were centrifuged and the pellets obtained were flash frozen.

#### 4.8.2. RNA Isolation

RNA isolation was done using Qiagen RNeasy RNA Isolation protocol. Briefly, RLT buffer plus was added to cell pellets and vortexed for 30 s. The homogenized lysate was transferred to a gDNA eliminator spin column and placed in a 2 mL collection tube. This was followed by centrifugation for 30 s at 10,000 RPM. The flow-through was saved and the column was discarded. 600 µL of 70% ethanol was added to the flow through and mixed well by pipetting. 700 µL of sample was then transferred to an RNeasy spin column and placed in a 2 ml collection tube. This was centrifuged at 10,000 RPM for 15 s, then the flow through was discarded. 700 µL of buffer RW1 was added to the RNeasy mini spin column and centrifuged at 10,000 RPM for 15 s followed by discarding the flow through. 500 µL of buffer RPE was added to the RNeasy spin column and centrifuged at 10,000 RPM for 15 s followed by discarding the flow through. 500 µL of buffer RPE was added to the RNeasy spin column and centrifuged at 10,000 RPM for 2 min. The RNeasy spin column was placed in a new 1.5 mL collection tube. 45 µL of RNase free water was added directly to the spin column membrane. Centrifugation was done at 8000 g for 1 min to elute the RNA. The final step was repeated to increase RNA eluate.

#### 4.8.3. RNA Quality Determination

The concentration and quality of RNA (RIN number) was determined using both a plate reader and a nucleic acid tapestation. The Gen 5 program was used to analyze RNA isolation and purification. Following purification, RNA-seq samples were shipped to the Novogene facility in Sacramento, CA and subjected to RNA-SEQ analysis.

In the Novogene facility, SMARTer Stranded V2 and NEB Small RNA Kits were used as PREP kits for the samples, followed by RNA quality control to validate the quality of RNA samples. Electrophoresis assay was performed to measure RNA concentration, area and RNA integrity number (RIN). RNA-Seq analysis was performed (Illumina Platform PE150), involving Library preparation. Long-coding RNA (lncRNA) and circular RNA (circ RNA) were subjected to raw data/sample & analysis which involved the following steps: (i) alternative splicing (AS) quantification and differential expression analysis (only for grouping with control samples), (ii) transcript Assembly, (iii) filtering of candidate lncRNA, (iv) co-location prediction of lncRNA and mRNA, (v) co-expression prediction of lncRNA and mRNA, (vi) gene ontology (GO) enrichment analysis, (vii) pathway enrichment analysis, (viii) transcription factors functional annotation analysis, (ix) protein-protein interaction analysis, (x) length distribution of circRNAs, (xi) distribution of circRNA on the chromosomes, (xii) transcript assembly, (xiii) filtering of candidate lncRNA, (xiv) gene ontology (GO) enrichment analysis, and (xv) Kyoto Encyclopedia of Genes and Genomes (KEGG) pathway enrichment analysis

Small RNA-seq was performed (Illumina Platform SE50) involving library preparation (small RNA library & seq 20 M raw reads/sample & analysis) which had the following steps: (i) identification of known miRNA, (ii) non-coding transcripts annotation, (iii) repeat sequence annotation, (iv) exon and intron annotation, (v) novel miRNA prediction, (vi) gene ontology, (GO) enrichment analysis, (vii) KEGG analysis, and (viii) pathway enrichment analysis.

#### 4.8.4. Ingenuity Pathway Analysis (IPA)

Various cell line/culture conditions were compared to one another to determine SMC generation. SRNA and lnc RNA data from novogene RNA-Seq analysis were uploaded to IPA. Genes related to adipose stem cell differentiation and vascular smooth muscle cell proliferation/differentiation/contraction were tracked from the RNA-Seq data. A differential core analysis was done to compare the specific mRNA/circRNA/miRNA expression between two cell line/medium formulations (A1PT vs A1CC, A2PT vs. A2CC, A2PT vs. A1PT). Pathways/networks between various molecules related to adipose stem cell differentiation and vascular smooth muscle cell expression were analyzed.

##### Data Analysis

To perform statistical analysis on the Western blot data, the fold change was calculated based on the normalized intensity of each treatment with respect to either the hASMCs or the DM treatment depending on trial. For CD44, the fold change was relative to the DM treatment. For SMC markers, the fold change was relative to the hASMCs. A two-sample equal variance t-test with two tails was used to test for significance between treatment and control. Additonally, *p*-values less than 0.05 were regarded as significant and denoted by *, while *p*-values less than 0.01 were denoted by **.

## 5. Conclusions

Our study focused on trying to determine the best time point and differentiation conditions for MSC differentiation to smooth muscle cells. We were able to determine that PT treatment was the best growth factor treatment for inducing differentiation in the shortest time (four days) for both bone marrow- and adipose-derived stem cells. Elastogenic VSMC obtained in this manner could be used in treating vascular disorders. This could be via (a) endovascular strategies, such as encapsulation in various tissue-engineered vascular grafts (TEVG) to develop into vascular tissue when implanted in vivo, or (b) perivascular strategies such as delivery of elastogenic VSMC or its secretome by cell delivery vehicles such as microbeads or nanoparticles into the site of injury. Our results also indicated that age and stem cell origin influenced MSC differentiation to SMC. However, we must state that, in this study, it was not possible to conclusively establish the differentiation capacity of MSCs toward the myogenic lineage in dependence of a given donor characteristic, as extensive tests with larger number of specimens and cell lines would be required to confirm our data. However, our results clearly showed that donor influence exists and thus, further characterization of the differentiation capacity of each single MSC line is required. In fact, differences were shown in the expression levels of marker molecules at the level of RNA. Future studies should look into cytokines present in the MSC secretome during differentiation to gain further insight into factors that accelerate vascular smooth muscle formation.

## Figures and Tables

**Table 1:** Application of three different growth factor-based DM treatments for a time point of four and seven days on three different bone marrow-derived stem cell lines. N/A in column 4 refers to results where none of the cell line samples in DM medium could show a higher expression of MYH11 than cell lines grown in CCM. Only PT (semi-batch) and PT treatment could elicit MYH II expression greater than CCM control in at least one trial.

Number of Trials	Cell Line	Time Period (Days)	Treatments with MYH11 Expression Greater than Cell Culture Medium Control
3	M1–30 Y Female Hispanic	4	PDGF + TGF β1 semi batch (1)
3	M1–30 Y Female Hispanic	7	PDGF + TGF β1 semi batch (1)
3	M2–18 Y Female African	4	PDGF + TGF β1 semi batch (1)
3	M2–18 Y Female African	7	N/A (3)
3	M3–21 Y Male Asian	4	PDGF + TGF β1 (1)
3	M3–21 Y Male Asian	7	N/A (3)

**Table 2:** Application of PDGF + TGFβ1 (PT) growth factor induced DM treatments to two adipose-derived cell lines (A1, A2) for a time point of four and seven days, respectively. PT (semi-batch) treatment could elicit MYH II expression greater than CCM control in at least one trial in all cases.

Number of Trials	Cell Lines (Adipose Tissue)	Time Period (Days)	Treatment with MYH II Expression Greater than CCM Control
3	A1–24 Y Female Caucasian	4	A1-PT (2)
3	A1–24 Y Female Caucasian	7	A1-PT (1)
3	A2–29 Y Female Native American	4	A2-PT (1)
3	A2–29 Y Female Native American	7	A2-PT (1)

**Table 3:** RNA-Seq data analysis among different ADSC cell line/medium formulations. A comparison between different formulations showing the presence of smooth muscle-specific long-coding RNA (lncRNA) and micro RNA (miRNA) in PT treated samples, compared to samples cultured in CCM.

	Gene Name	Significance	Expression Log Ratio	Result
A2PT vs. A2CC	actin alpha 2 (ACTA2)	Expressed in smooth muscle and aids in vascular contraction	3.758	Increased expression in A2PT
	miR-17-5p	Mesenchymal stem cell differentiation	2.130	
	miR-145-5p	Vascular smooth muscle cell differentiation	3.682	
A1PT vs. A1CC	transgelin (TAGLN)	Expressed in vascular and smooth muscles and is an early marker of smooth muscle differentiation.	3.272	Increased expression in A1PT
A2PT vs. A1PT	actin alpha 2 (ACTA2)	Expressed in smooth muscles and aids in vascular contraction	3.044	Increased Expression in A2PT Decreased Expression in A2PT
	miR-21-5p	Adipose mesenchymal stem cell differentiation	−3.523	

**Table 4:** RNA-Seq data analysis among different ADSC cell lines and treatments. A comparison between different treatments showing the presence of smooth muscle-specific circular RNA (Circ RNA) in PT treated samples compared to samples cultured in CCM.

	Gene Name	Significance	Result
A2PT vs. A2CC	CALD1	Regulates smooth muscle contraction	Increased expression in A2PT
	ROCK2	Regulates formation of actin stress fibers	
	PPP1R12C	Regulates the assembly of the actin cytoskeleton	
A1PT vs. A1CC	CALD1	Regulates smooth muscle contraction	Increased expression in A1PT
A2PT vs. A1PT	CALD1	Regulates smooth muscle contraction	Increased expression in A2PT
	PP1R12C	Regulates the assembly of the actin cytoskeleton	

## Data Availability

No dataset has been deposited in a repository. All generated data is included in the manuscript.
